# Supplementary figures and images for: Body Composition and Metabolic Dysfunction Really Matter for the Achievement of Better Outcomes in High-Grade Serous Ovarian Cancer
Source: Cancers (Basel). 2023 Feb 10;15(4):1156. doi: 10.3390/cancers15041156 (PMC9953877; doi:10.3390/cancers15041156)

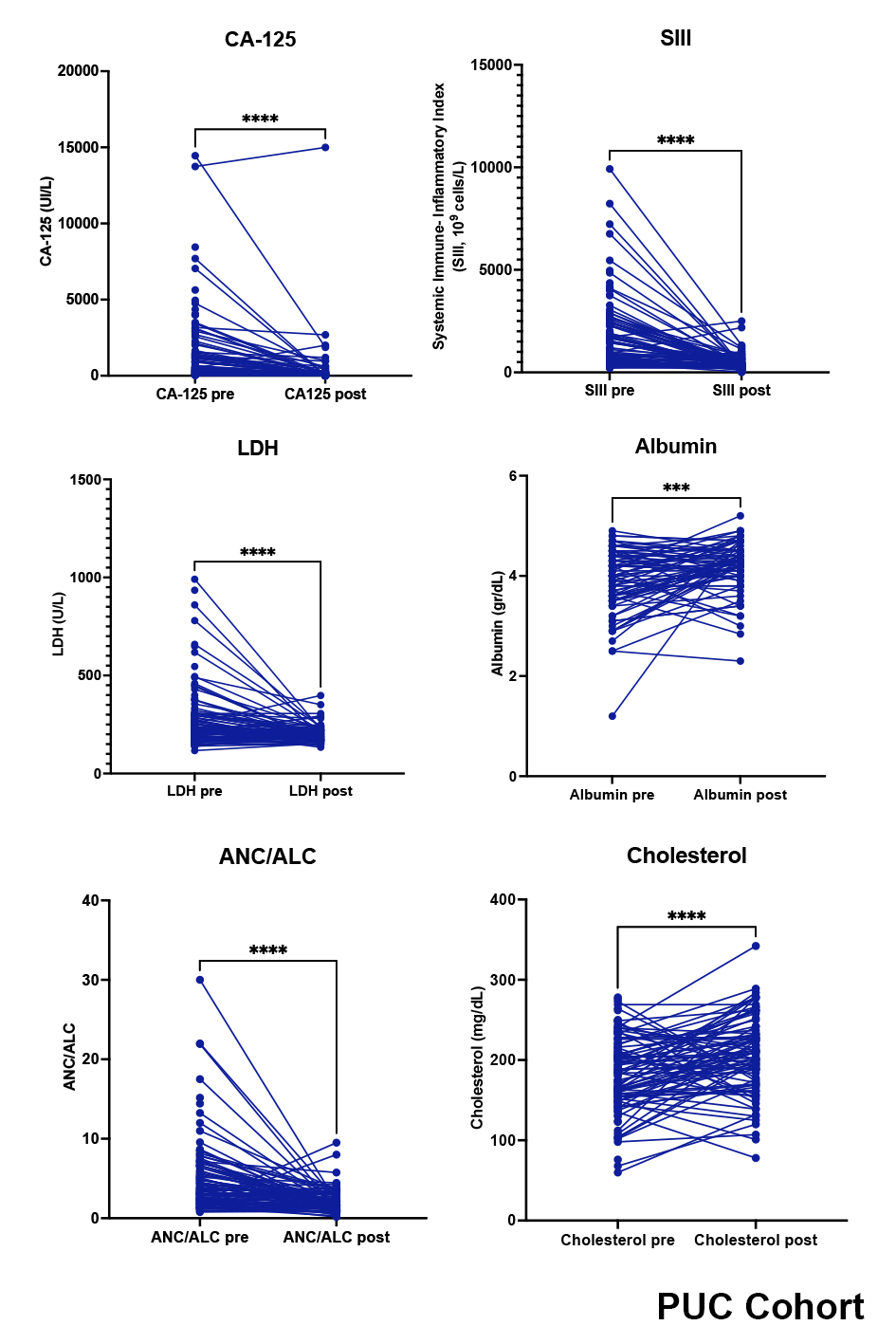

Supplement: Supplementary file 1 [file cancers-15-01156-s001.zip › cancers-2130992-supplementary/Suppl material cancers-2130992_version2/Suppl Fig 1 BC_HGSOC_Cuello_M_Cancers_new.tif]

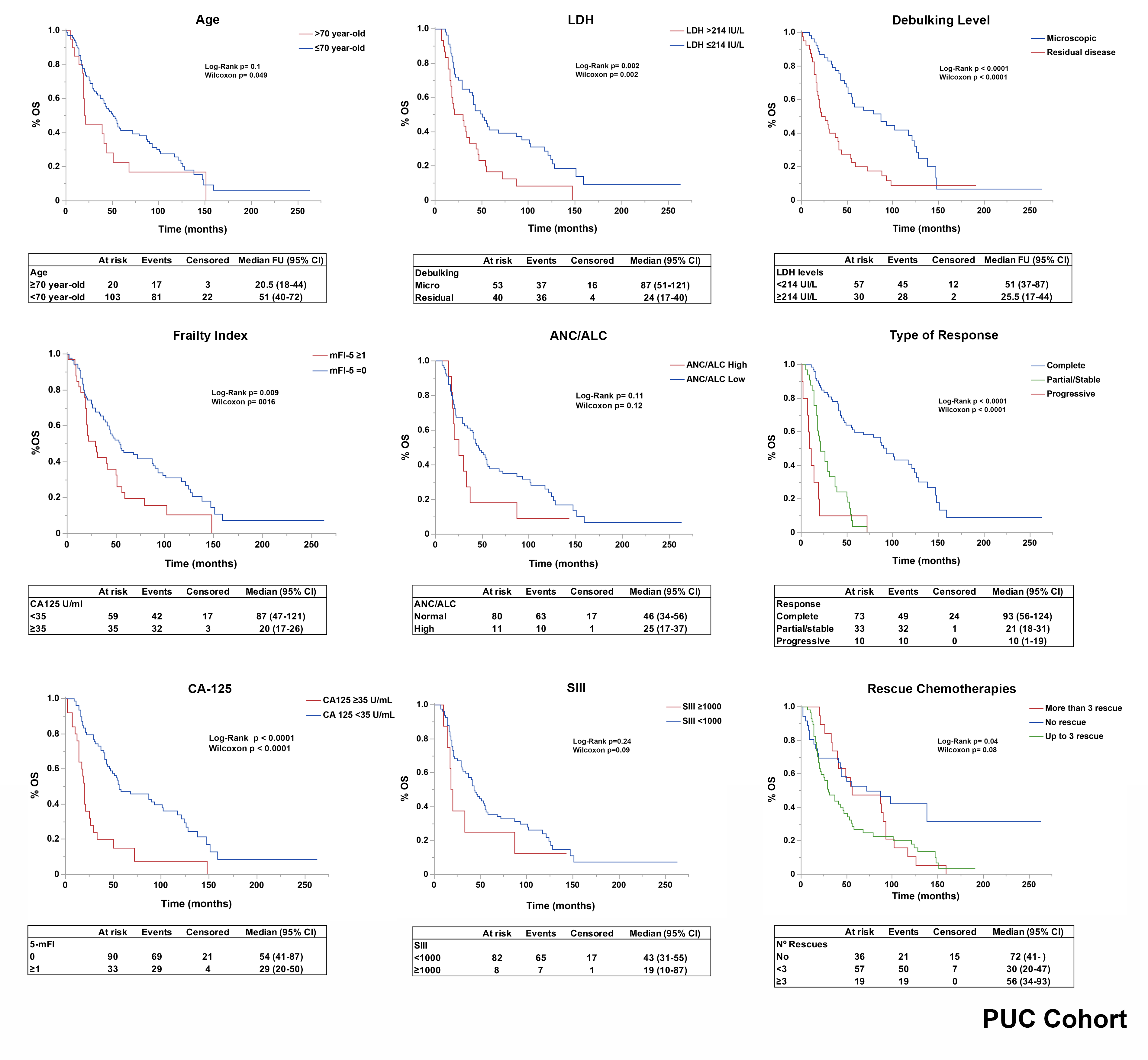

Supplement: Supplementary file 1 [file cancers-15-01156-s001.zip › cancers-2130992-supplementary/Suppl material cancers-2130992_version2/Suppl Fig 2 BC_HGSOC_Cuello_M_Cancers_new.tif]

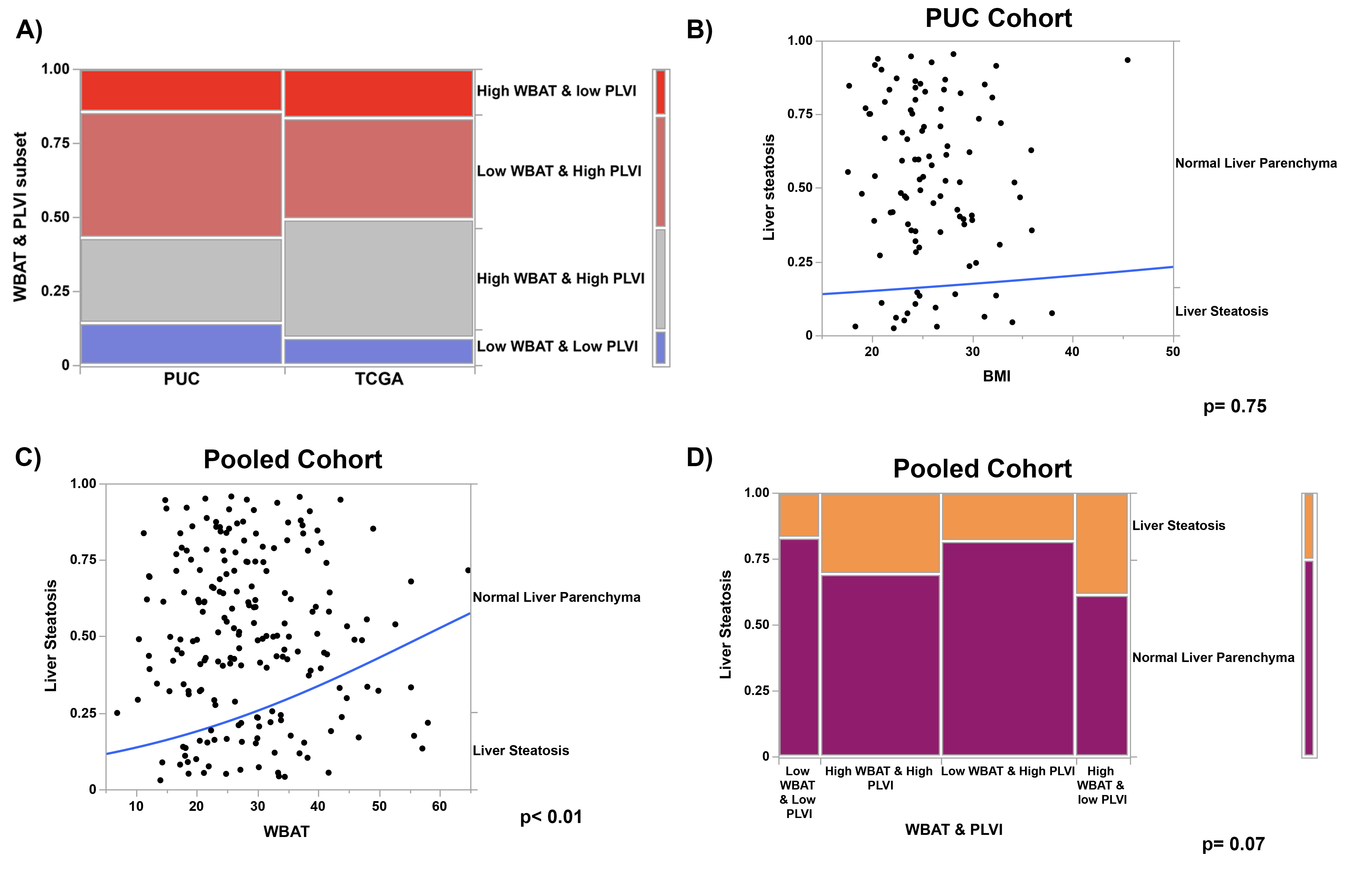

Supplement: Supplementary file 1 [file cancers-15-01156-s001.zip › cancers-2130992-supplementary/Suppl material cancers-2130992_version2/Suppl Fig 3 BC_HGSOC_Cuello_M_Cancers_new.tif]

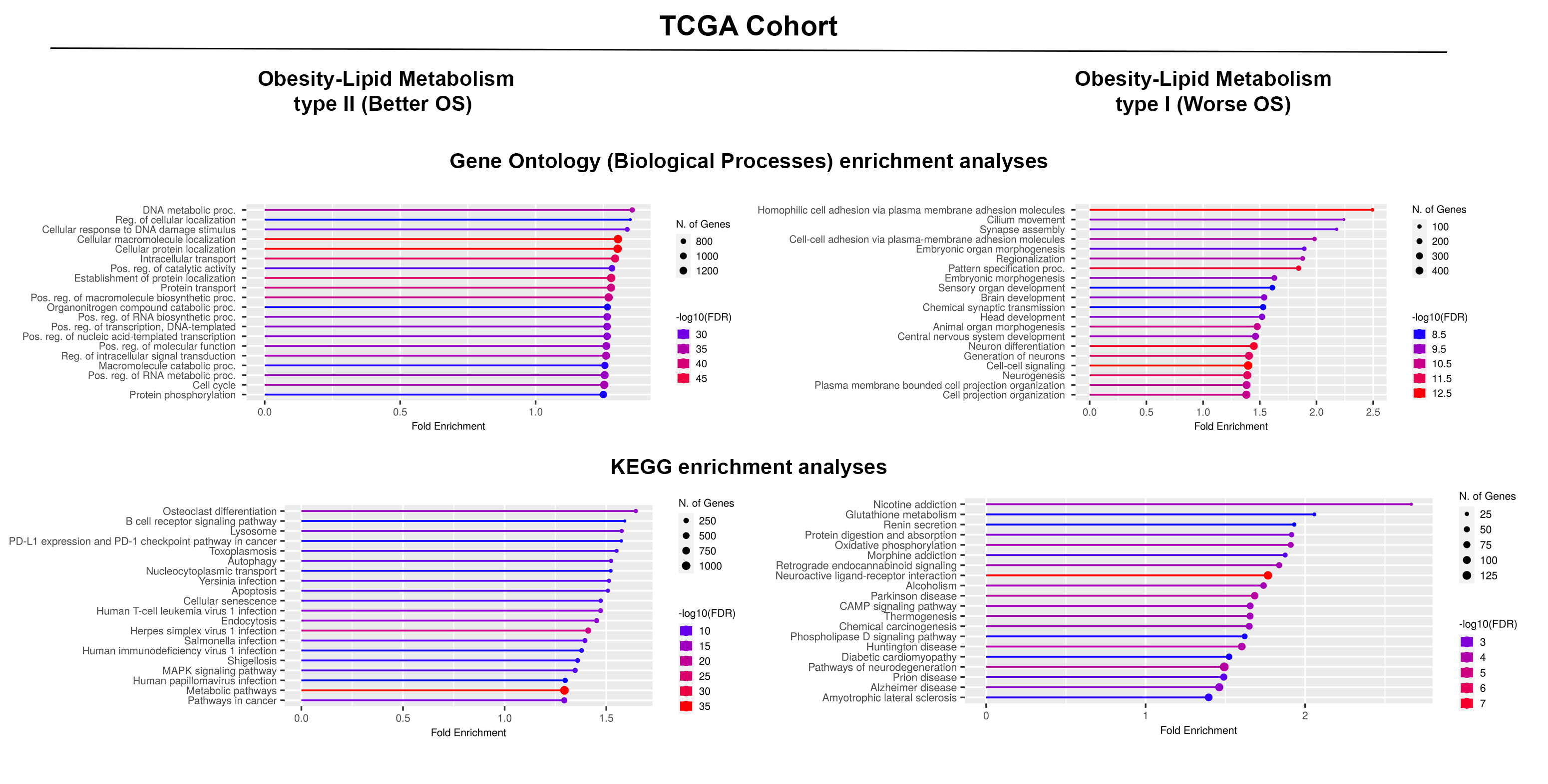

Supplement: Supplementary file 1 [file cancers-15-01156-s001.zip › cancers-2130992-supplementary/Suppl material cancers-2130992_version2/Suppl Fig 4 BC_HGSOC_Cuello_M_Cancers_new.tif]

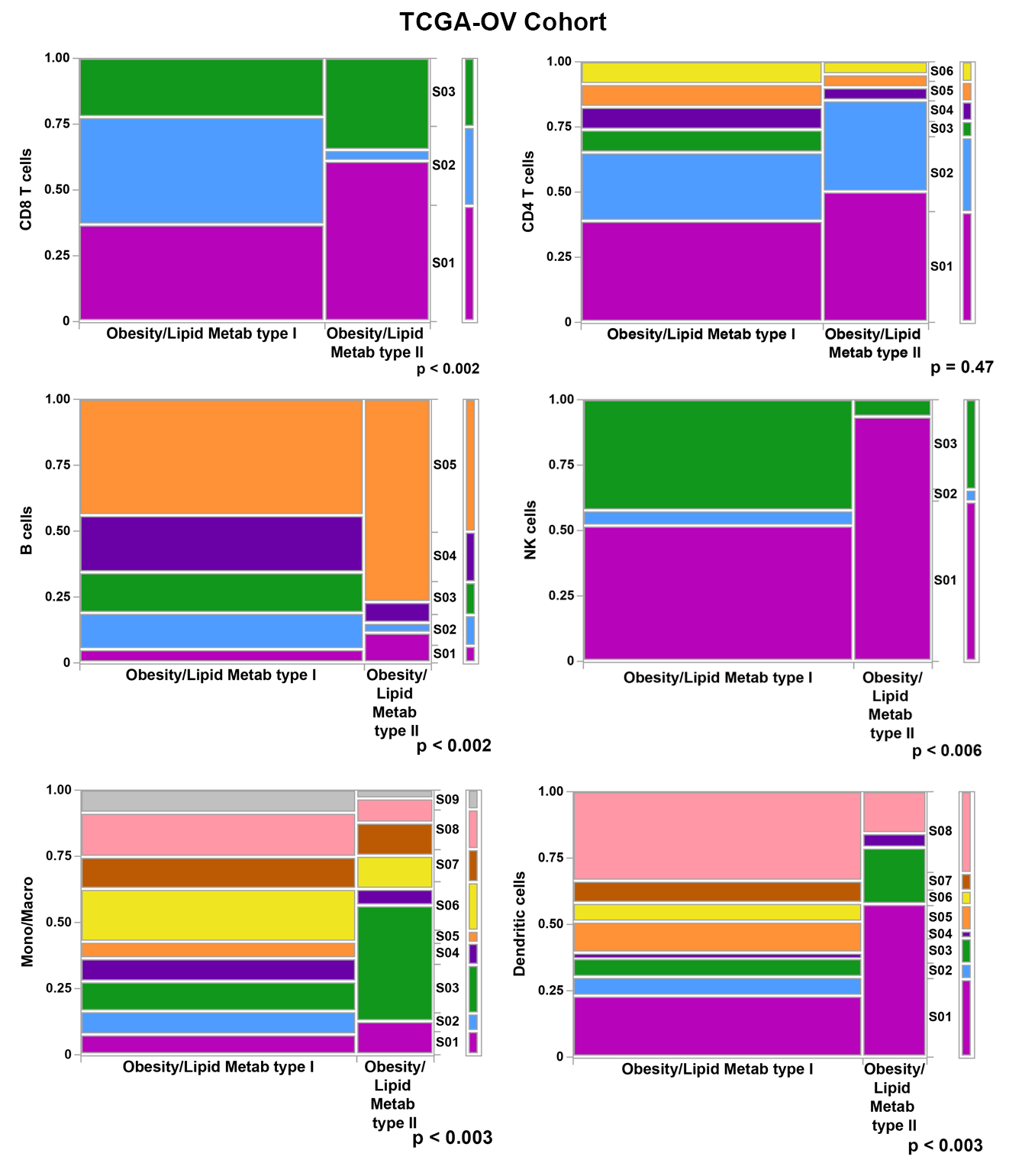

Supplement: Supplementary file 1 [file cancers-15-01156-s001.zip › cancers-2130992-supplementary/Suppl material cancers-2130992_version2/Suppl Fig 5 BC_HGSOC_Cuello_M_Cancers_new.tif]
